# Interventions to improve vaccine acceptance among rheumatoid arthritis patients: a systematic review

**DOI:** 10.1007/s10067-019-04430-7

**Published:** 2019-01-15

**Authors:** Vincent Gosselin Boucher, Ines Colmegna, Claudia Gemme, Sara Labbe, Sandra Pelaez, Kim L. Lavoie

**Affiliations:** 1 Department of Psychology, University of Quebec at Montreal (UQAM), CP 8888, Sucursale Centre-Ville, Montreal, Quebec H3C 3P8 Canada; 2 Montreal Behavioural Medicine Centre, Centre Intégré Universitaire de santé et services sociaux du Nord-de-l’Ile-de-Montréal (CIUSSS-NIM), Hôpital du Sacré-Coeur de Montréal Canada, Montréae, Canada; 3 Department of Medicine, Division of Rheumatology, McGill University, Montreal, Canada; 4 Department of Educational and Counselling Psychology, McGill University, Montreal, Canada

**Keywords:** Behavioral interventions, Rheumatoid arthritis, Systematic review, Vaccination

## Abstract

**Introduction/objective:**

National guidelines emphasize the importance of annual immunization for patients living with rheumatoid arthritis (RA), but vaccination rates remain suboptimal in this population. Evaluating the efficacy of patient and/or provider-targeted interventions to improve vaccination uptake among RA patients could inform practice.

**Methods:**

We conducted a systematic review (SR) to examine the efficacy of interventions (exposure) aiming to improve vaccination uptake in patients with RA (outcome). English and French language, peer-reviewed interventional studies to improve vaccination rates in RA patients published between 2009 and 2018 were included.

**Results:**

The search yielded a total of 450 records. Five articles met inclusion criteria. All interventions focused on changing provider behavior using some form of vaccination reminder as the primary intervention strategy, though only two studies reported provider prescribing behavior as an outcome (which was 4% and 58%). Overall, studies varied greatly regarding intervention delivery mode (e.g., educational sessions, e-mail reminders, best practice alerts), and behavior change techniques used to encourage providers to prescribe vaccination (e.g., feedback and monitoring, shaping knowledge, self-regulation). For influenza, pneumococcal and herpes zoster, post-intervention (mean 12–16 months follow-up) vaccination rates increased by a mean of 16.6% (± 15.4%).

**Conclusions:**

Interventions to enhance vaccine uptake in RA focused almost exclusively on improving provider prescription of vaccines using reminder-type interventions. Although effective in improving vaccination rates, those studies used heterogeneous interventions and behavior change techniques. Few studies measured provider prescribing behavior as an outcome. Future studies targeting providers should measure relevant provided-related outcomes and their impact on patient outcomes, to determine overall efficacy.

Rheumatoid arthritis (RA), the most common musculoskeletal inflammatory disorder worldwide [1], affects approximately 1% of the population [2]. RA is a chronic systemic autoimmune inflammatory disease that primarily manifests with synovitis, usually polyarticular. This disease is more common among women (F/M ratio = 2:1), and the lifetime risk of developing RA in adulthood is 3.6% for women and 1.7% for men [3]. From a public health perspective, the costs associated with RA are substantial and estimated at > $39.2 billion annually in the USA [4].

A substantial burden of RA relates to the increased morbidity and mortality associated with infectious diseases [5]. RA patients have higher risk of two major vaccine-preventable respiratory organisms: influenza and pneumococcus [6]. Further, people living with RA are two times more likely to develop medical complications that frequently require hospitalization due to those respiratory diseases than age-matched healthy controls [7, 8]. This highlights the need to target RA patients for vaccination [5]. Despite current recommendations that identify RA patients as a high-priority group for vaccination [9], vaccination coverage among RA patients is suboptimal [10]. Reported immunization rates range between 25–90% for influenza and 17–62% for pneumococcus [10–15]. This is often below the target proposed by the World Health Assembly for seasonal flu vaccination coverage in at-risk populations which is 75% [16].

Improving vaccination rates is therefore a public health priority as it may enhance protection in adults living with RA as well as the community at large. Several factors have been associated with low vaccination rates in RA. Patient-related factors include high perceived vaccine risk and low perceived efficacy [17]. Provider-related factors include the failure to advocate for and prescribe vaccines to RA patients [14]. Although this topic is of high interest from a public health perspective, to our knowledge, no study has systematically reviewed the evidence on the nature and efficacy of interventions aiming to improve vaccination uptake among RA patients. Thus, the purpose of this review was to fill this knowledge gap by assessing the efficacy of existing interventions targeting either patients and/or health care providers aiming to improve vaccine uptake among RA patients. Since vaccination acceptance is a health-related behavior, we were particularly interested in behavioral interventions addressing vaccine uptake. Also, we aim to dissect the specific components (e.g., content, format, structure) of the most efficacious interventions.

## Methods

The PRISMA checklist was followed to ensure transparent and comprehensive reporting throughout the systematic review [18]. The review was registered with the International Prospective Register of Systematic Reviews (PROSPERO: CRD42018103564) [19].

## Inclusion and exclusion criteria

Studies reporting the results of interventions to improve vaccination uptake in RA were included. More specifically, studies on behavioral interventions [20] targeting providers and/or patients to enhance vaccination uptake among RA patients. There was no restriction on vaccine type (e.g., influenza, pneumococcal, herpes zoster). Only English or French publications in peer-reviewed journals reporting pre- and post-intervention measures of vaccination (e.g., vaccination rates) were included. Studies assessing interventions to improve vaccination rates in the general population or in chronic diseases other than RA (e.g., cancer, pulmonary disease) were excluded.

## Search strategy and review process

PUBMED, PsychINFO, SCOPUS, and Cochrane searches up to July 25th 2018 were conducted. The keyword terms used were: “vaccination” AND “rheumatoid arthritis” AND “behaviour change”, “behavior change”, “motivational interviewing”, “motivation communication”, “counseling”, “counselling”, “behavioral”, “intervention”.

**Fig. 1:**
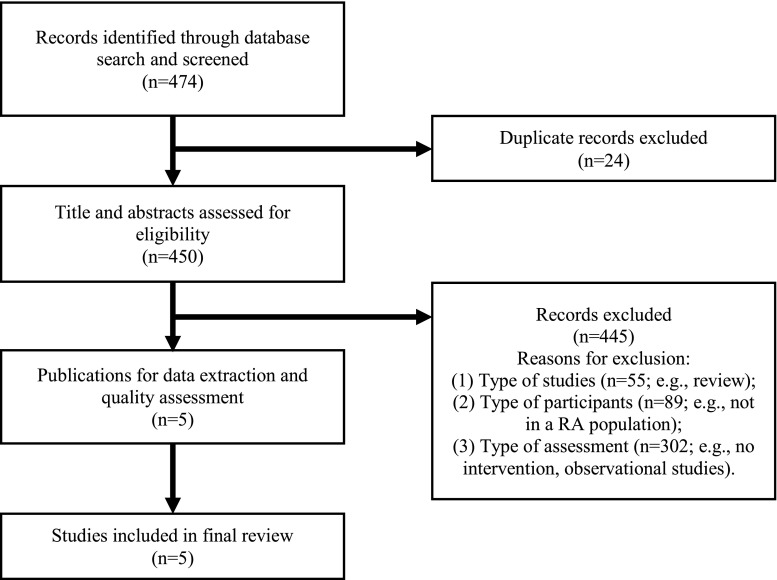
Flow diagram

Reference lists of selected publications were screened to identify additional studies (see Table 1). This search process generated 450 unique and potentially eligible studies. As seen in Fig. 1, only five articles fulfilled the inclusion/exclusion criteria [21–25]. The following information was extracted from those five studies: participants (number and type of provider and RA patients included); outcomes of interest (e.g., vaccination rates, provider behaviors around vaccination prescribing); intervention characteristics (e.g., type, timing, structure, components of the intervention, follow-up period); comparison group characteristics (when applicable)

**Table 1:** Intervention study details

Author [ref]	Study design	Outcome and target	Provider sample	RA patients (*n*)	Intervention	Comparison group	Post-evaluation
*Interventions targeting providers*							
Ledwich et al. [21]	Pre-post quasi experimental intervention design	Vaccination rates (patient) and Documentation of prescription (provider)	Health care providers; physician, fellow, resident, or nurse practitioner (n not reported)	758	Electronic Health record (EHR) best practice alert (BPA)	None	Did not report
Desai et al. [22]	Cluster, Controlled trial; Quality improvement intervention strategy	Vaccination rates (number of patients up to date) (patient)	Rheumatologists (*n* = 14)	3717	Point-of-care paper reminder forms	21 Rheumatologists	Assessed monthly (for a median of 16 months)
*Interventions targeting providers and patients*							
Baker et al. [23]	Quasi-experimental: Pre-post system-level intervention for quality improvement	Vaccination rates (patient)	Rheumatologists and primary care physician (*n* = 8)	1255	Reminders to prescribe vaccination, performance feedback to physicians and letters to patients	None	Assessed monthly for 12 months
Sheth et al. [24]	Pre-post quasi-experimental quality improvement intervention design	Vaccination rates (patient) and documentation rate (provider)	Physicians and staff (n not reported)	1554	Real-time electronic medical record (EMR) based alert system (BPA), coupled with patients and staff education and physician feedback and interval assessment	None	Did not report
Broderick et al. [25]	Quasi-experimental, Pre-post multimodal intervention	Decrease frequency of any missed opportunities for vaccination and vaccine attitude (0–100) (provider)	Rheumatologists (n not reported)	197	Multimodal intervention using education session, EMR-based alerts and personalised e-mail reminders for patient	None	Assessed each 3 months for 12 months
Author [ref]	Pre-intervention measures (HCPs)		Post-intervention measures (HCPs)		Pre-intervention measures (patients)	Post- intervention measures (patients)	
*Interventions targeting providers*							
Ledwich et al. [21]	NA		NA		Influenza vaccination rates: 47%; Influenza documentation: 47%; Pneumococcal vaccination rates: 19%; Pneumococcal documentation: 19%	Influenza vaccination rates: 65%; Influenza documentation: 67%; Pneumococcal vaccination rates: 41%; Pneumococcal documentation: 45%	
Desai et al. [22]	NA		NA		Intervention group rates of patients who were up-to-date for pneumococcal: 67.6%; Control group rate: 52.3%	Intervention group rates of patients who were up-to-date for pneumococcal: 80% (*p* < 0.006); Control group rate: 52.0% / (pre-post: *p* = 0.941)	
*Interventions targeting providers and patients*							
Baker et al. [23]	Not reported		Action rate^a^: first 2 months = 45–57%; months 3 to 5 = low of 38%; months 6–12 = 42–58%		Influenza: Ever received (90.2%); in previous season (79.4%); Pneumococcal: Ever received (28.7%); Herpes Zoster: Ever received (2.5%)	Influenza: Ever received (86.1%), in previous season (78.2%); Pneumococcal: Ever received (45.8%); Herpes Zoster: Ever received (4.5%)	
Sheth et al. [24]	Not reported		“Among 1002 patients for whom the BPA appeared, 581 (58%) resulted in either a vaccination (252; 43% vaccinated, 21; 4% vaccine prescribed) or documentation of reasons the vaccine was not prescribed (308; 53%)”		Herpes Zoster vaccination rates: 10.1%; vaccines documentation rates: 28%	Herpes Zoster vaccination rates: 51.7% (p < 0.0001).; vaccines documentation rates: 72.9% (*p* < 0.0001).	
Broderick et al. [25]	NA		NA		Frequency of any missed influenza vaccination: 47%; vaccination attitude: 50 ± 9	Frequency of any missed influenza vaccination: 23% (*p* < 0.001); vaccination attitude: 51 ± 9	

^a^The proportion of patients who were seen by their rheumatologist who had: a vaccination given, a historical vaccination documented, or a documented medical or patient reason for not giving a vaccination

## Study quality

Study quality was assessed by two independent evaluators using the Downs and Black checklist, a 27-item rating system assessing the methodological quality of randomized and non-randomized interventions [26]. This checklist helps reviewers identify the strengths and weaknesses of the methodological design and reporting quality (e.g., randomization, blinding, reporting bias) of each reviewed study. Inter-rater agreements and kappa coefficients [27] were calculated with a 95% confidence interval using the two raters’ scores (V.G.B. and S.L.) for each study included in this review. This was done at the data extraction phase (86%, kappa = 0.7) and after study inclusion (study quality, 89%, kappa = 0.7). The inter-rater agreement score for the first round of eligibility assessment by titles and abstracts screening was also very high (90%).

## Results

### Study characteristics

Study characteristics are summarized in Table 1. Out of five studies, three targeted providers and two providers and patients. The number of providers participating in the studies was generally small (8–35) or was not reported (*n* = 3 studies) [21, 24, 25]. Patients in the studies ranged from 197 to 3717. The identified objective of the intervention was “improving vaccination rates” (in patients) [21–24], “reducing the frequency of any missed opportunities for vaccination” (in providers) [25], and “increase the documentation of prescriptions (in providers)” [21, 24]. Three studies reported follow-up periods [22, 23, 25]. Time to post-intervention evaluations was generally 1 year, with two studies assessing patient vaccination rates monthly, for a median follow-up period of 12 [23] to 16 months [22].

### Interventions’ characteristics

Intervention target, duration, content, and delivery mode varied considerably. All studies included interventions that targeted provider behavior to increase vaccination rates, and all provider interventions involved some form of vaccination reminder to discuss or prescribe vaccination as the primary intervention, including Electronic Health Record (EHR) or Electronic Medical Record (EMR) alerts and Point-of-care paper reminder forms. Only three studies also targeted patients and included education [24] or letters/e-mail reminders [23, 25] to improve vaccination rates. Intervention components varied considerably between studies. For this review, we defined an intervention component as any intervention strategy, implicitly or explicitly stated by the original study, as summarized by Michie’s Taxonomy [20]. To achieve an adequate comparison of each intervention’s content, the first (V.G.B.) and second (S.L.) reviewers identified their unique components and linked them to the Behavior Change Techniques (BCT’s) identified by Michie and colleagues [20]. Among provider-targeted interventions, we identified a total of three BCTs used across the two studies and included: (1) adding objects to the environment (i.e., paper reminder forms, 1/2 studies) [22], (2) monitoring behavioral outcomes (EHR best practice alerts, 1/2 studies) [21], and (3) pharmacological support (provided vaccination documentation or advice to get vaccinated, 2/2 studies) [21, 22]. Among patient-targeted interventions, we identified a total of five BCTs including (1) adding objects to the environment (i.e., paper reminder forms, 2/3 studies) [23, 25], (2) providing feedback on behavior (i.e., performance feedback, 2/3 studies) [23, 24], (3) monitoring behavioral outcomes (EMR-based alert, 2/3 studies) [24, 25], (4) instruction on how to perform the behavior (educational session (s) with follow-up assessments; 2/3 studies) [24, 25], and (5) pharmacological support (provided vaccination documentation or advice to get vaccinated, 3/3 studies) [23–25] (see Table 2).

**Table 2:** Intervention components

Behavior Change Technique Taxonomy (Michie et al. 2013)	Ledwich et al. [21]	Desai et al. [22]	Baker et al. [23]	Sheth et al. [24]	Broderick et al. [25]
*Interventions targeting providers only*					
Feedback and monitoring (2.) Monitoring of outcomes behavior without feedback (2.5)	√				
Regulation (11.) Pharmacological support (11.1)	√	√			
Antecedents (12.) Adding objects to the environment (12.5)		√			
*Interventions targeting providers and patients*					
Feedback and monitoring (2.) Feedback on behavior (2.2)			√	√	
Monitoring of outcomes behavior without feedback (2.5)				√	√
Shaping knowledge (4.) Instruction on how to perform the behavior (4.1)				√	√
Regulation (11.) Pharmacological support (11.1)			√	√	√
Antecedents (12.) Adding objects to the environment (12.5)			√^a^		√^a^

^a^Only the patients

Few studies provided information on the structure of the intervention. Only two studies provided formal education sessions [24, 25], the content of which varied: Broderick et al. focused on the rationale, efficacy, and recommended uses of vaccination [25], while Sheth and colleagues [24] had providers engage in small group discussions to address concerns, clarify misconceptions, and update recommendations regarding vaccination [24]. The interventions were brief at only one [24] or two [25] sessions. None of these studies reported the duration (e.g., total minutes/hours/days) of the sessions, trainers’ qualifications, trainee attendance or participation, or training program fidelity.

### Interventions’ results

Due to the high heterogeneity of intervention components and the lack of standardized reporting of outcomes, we were unable to perform a formal meta-analysis. However, the interventions included in this review were shown to be effective (see Table 1). For the three types of vaccines targeted (influenza, pneumococcal, and herpes zoster), pre-intervention vaccination rates ranged from 47 to 79.4% for influenza vaccine [21, 23], 19 to 28.7% for pneumococcal vaccine [21, 23], and 2.5 to 10.1% for herpes zoster vaccine [21, 23]. Post-intervention (12-month follow-up) vaccination rates increased by a mean of 8.4 ± 13.6% for influenza, 19.6 ± 3.5% for pneumococcal, and 21.8 ± 28.0% for herpes zoster vaccine. These were statistically significant improvements. Broderick and colleagues measured the frequency of any missed influenza vaccination pre-intervention (47%) and post-intervention (23%; *p* < 0.01). In the only controlled study with a comparison group, rheumatologist did not receive paper reminder [22], pre-intervention rates of pneumococcal vaccination were 67.6% in the intervention group and 52.3% in the control group. Post-intervention (median of 16-month follow-up) pneumococcal vaccination rates were 80% (+ 12.3, *p* < 0.01) in the intervention group and 52% (− 0.3, *p* = 0.09) in the comparison group.

Although all studies targeted providers, only two reported post-intervention measures of prescription behaviors [23, 24] and none baseline frequencies. Baker and colleagues [23] reported the “action rate” of providers (the proportion of patients who were seen by their rheumatologist who had a vaccination given, a historical vaccination documented, or a documented medical or patient reason for not giving a vaccination), which varied from 38 to 58% during the 12 months of implementation. The second study reported the proportion of patients that were vaccinated (43%) or for whom a vaccine was ordered (4%) or for whom a reason for not getting vaccinated was documented (3%: physician deferred [27%] or patients declined [73%]). Also, two studies reported pre-post vaccination prescription documentation rates with a mean increase of 30% [21, 24]. Finally, one study evaluated vaccination attitudes among RA patients (the Vaccine Attitudes Questionnaires, score 0–100) [25] and showed no change from pre-post intervention (50 ± 9 to 51 ± 9; *p* = 0.58).

### Study quality

The methodological quality of the studies varied considerably (Downs and Black [26] checklist score range: 11–15) with an average score of 13 out of 28 denoting moderate quality. None of the studies received an excellent rating (26–27) or scores below 10 that are considered of poor quality (see appendix for scores). The low-quality scores of the studies were mostly attributable to non-randomized designs and the lack of comparison groups.

## Discussion

This study reviewed the existing literature addressing the impact of behavioral interventions on vaccine uptake among RA patients. Overall, few studies have been conducted to date targeting this topic in this population. Reminder-type interventions were the most commonly used interventions to improve provider prescription of vaccines and vaccine uptake among RA patients. Despite the narrow focus on primarily provider-targeted interventions involving reminders to vaccinate, intervention strategies (e.g., Electronic Medical Record [EMR] alerts, point-of-care paper and electronic reminders, practice feedback) were heterogeneous, making impossible to conduct a formal meta-analysis to assess the overall magnitude of their effects. Although all studies reported improvements in vaccination rates among RA patients, none actually measured provider prescription behavior pre- and post-intervention, which makes it difficult to determine the true efficacy of the intervention. When conducting behavioral interventions, measuring behavioral mediators (e.g., vaccination prescription by providers), it is key to assess the interventions’ hypothesized mechanism(s) of action on clinical outcomes [28] (e.g., vaccine uptake among patients). All studies reviewed focused on changing provider vaccination prescribing behavior as the primary means of increasing vaccination rates, but all failed to systematically assess pre- and post-intervention rates of vaccination prescription. Investigators in this area are encouraged to measure changes in these intermediate behavioral targets, as well as the association between these changes and clinical outcomes, in order to determine the true effectiveness of these interventions. Finally, generalizability of the reminder-type interventions is limited by the poor methodological reporting of intervention details including intervention schedule (number of sessions), dose (duration of intervention), or educator details. Failure to report this information does not allow study replication, limiting their value and potential impact.

Only three studies included interventions that also targeted patients. However, the failure to measure provider prescription behavior makes it impossible to know which intervention targets were more effective: those targeting the providers or those targeting providers and patients, as only patient-level outcomes (vaccination rates) were measured.

In general, the results of this review are consistent with similar studies using reminder-based interventions targeting physicians to improve vaccination rates in the context of vaccination [29, 30]. However, they were not consistent (no significant change post-intervention) with one study that provided letters to physicians with the aim of reducing missing vaccination opportunities to improve MMR vaccination among children [31]. It is possible that when vaccines target children, where parents are making decisions on their behalf, provider reminders that do not address the complex concerns of parents are not as effective. Additionally, the results of the present review were also consistent with studies using reminder-based interventions targeting patients with other diseases. For example, two studies using brochure reminders [32] or mailed reminders [33] showed significant increases in vaccination rates post-intervention among patients at high risk of infections (ranging from 1.6 to 6% higher rates). However, not all studies using reminder-based interventions with patients at risk of infection were effective [34, 35]. Finally, two studies failed to observe increases in MMR immunization rates in association with text messages targeting pregnant women [35] and telephone reminders with home visits among parents [34]. Once again, it is possible that reminder-based interventions are insufficient to address the complex concerns of parents around immunization of their children.

### Limitations

This review is limited by the low number of studies meeting inclusion criteria, the variety of their interventions and the methodological heterogeneity that precludes conducting a formal meta-analysis, and the inclusion of studies of generally moderate methodological quality. Key limitations of the studies included in this review include the lack of randomized designs and absence of comparison groups, low sample sizes, inadequate reporting of methodological details (duration, dose), and the failure to measure targeted behavioral outcomes (vaccination prescription) among providers. An additional missing component of all studies was the lack of reported stakeholder involvement in intervention protocol development. The integration of stakeholders (e.g., patients, HCPs, administrators) is encouraged to identify research priorities, define relevant outcomes, and help clinical translation/implementation [36, 37].

### Conclusion

This review highlights the paucity of research on the efficacy of interventions designed to improve vaccination uptake among RA patients, despite the sub-optimal vaccination rates and the fact that RA patients are a high-risk population. Furthermore, this review indicates that all interventions to date have focused on changing provider behavior to improve vaccination rates, without interventions targeting specifically patients’ factors such as perceived lack of vaccine efficacy or concerns over side effects. Accepting to be vaccinated is a complex behavior that relies upon both provider and patient factors that will likely be inadequately addressed in interventions focusing exclusively on one or the other. Consequently, future studies should develop and test interventions targeting both provider and patient behavior. One such intervention may be motivational communication (MC), which help providers educate, motivate, and enable patients to engage in appropriate and beneficial self-management behaviors to improve chronic disease outcomes [38, 39]. This approach involves training providers in evidence-based behavior change techniques that focus on shared-decision-making that links patients’ health objectives (e.g., higher number of pain-free days, improved mobility) to engaging in positive health behaviors (e.g., vaccination). Interventions using MC-based strategies have been shown to be associated with improvements in a wide range of health behaviors (e.g., medication adherence, physical activity, and exercise) and clinical outcomes (e.g., patient health). The extent to which MC may be efficacious for improving vaccination rates among RA patients remains to be determined, but could be promising to address this complex behavior.
